# A High-Performance and Flexible Architecture for Accelerating SDN on the MPSoC Platform

**DOI:** 10.3390/mi13111854

**Published:** 2022-10-29

**Authors:** Meng Sha, Zhichuan Guo, Yunfei Guo, Xuewen Zeng

**Affiliations:** 1 National Network New Media Engineering Research Center, Institute of Acoustics, Chinese Academy of Sciences, No. 21, North Fourth Ring Road, Haidian District, Beijing 100190, China; 2 School of Electronic, Electrical and Communication Engineering, University of Chinese Academy of Sciences, No. 19(A), Yuquan Road, Shijingshan District, Beijing 100049, China

**Keywords:** SDN, network acceleration, FPGA, reprogrammable hardware

## Abstract

Software-defined networking has been developing in recent years and the separation of the control plane and the data plane has made networks more flexible. Due to its flexibility, the software method is used to implement the data plane. However, with increasing network speed, the CPU is becoming unable to meet the requirements of high-speed packet processing. FPGAs are usually used as dumb switches to accelerate the data plane, with all intelligence centralized in the remote controller. However, the cost of taking the intelligence out of the switch is the increased latency between the controller and the switch. Therefore, we argue that the control decisions should be made as locally as possible. In this paper, we propose a novel architecture with high performance and flexibility for accelerating SDN based on the MPSoC platform. The control plane is implemented in the on-chip CPU and the data plane is implemented in the FPGA logic. The communication between the two components is performed using Ethernet communication. We design a high-performance TCAM based on distributed RAM. The architecture employs a pipeline design with modules connected via the AXI Stream interface. The designed architecture is flexible enough to support multiple network functions while achieving high performance at 100 Gbps. As far as we know, the architecture is the first proposed in the design of a 100 Gbps system.

## 1. Introduction

The ever-increasing network demands of users make today’s network environment more and more complex, which makes traditional IP networks show their disadvantages such as the difficulty of maintaining and scaling a multi-device network [1]. These disadvantages have promoted the emergence and development of new network architectures. However, it has been observed that it is not easy to validate these novel network architectures in existing legacy networks due to the limitations of conventional rigid network equipment. The difficulty for conventional network devices to adapt quickly to changes in the network environment has given rise to the need for “network programmability” [2,3,4].

Several new network architectures are being developed to enable the programmability of networks such as network function virtualization (NFV) [5] and software-defined networking (SDN) [6]. SDN separates the data plane from the control plane and the network intelligence is stripped from network devices and placed on the controller. The data plane is only responsible for implementing the forwarding behavior of a network. Various types of devices are utilized to implement the data plane, including central processing units (CPUs) [7,8,9], and application-specific integrated circuits (ASICs) [10]. ASICs can be used to achieve optimal performance in the data plane but are designed for specific networking applications and cannot be reprogrammed. CPUs can be used to provide the best flexibility. It is easy to change the forwarding behavior of the data plane by running different software applications. Such flexibility, however, comes at the cost of performance. Even with the help of kernel-bypassing high-performance packet I/O frameworks such as DPDK [11] and NetMap [12], it is still impossible to achieve the line rate for forwarding 64-byte packets [13]. Even if high performance is achieved by using multi-core CPUs, a lot of CPU resources are wasted.

In recent years, field programmable gate arrays (FPGAs) have been widely used both in academia and industry [14,15,16,17] because they can achieve higher performance than CPUs while having programmability comparable to ASICs. In traditional heterogeneous SDN networks, the FPGA acts as a dumb forwarding device that simply accelerates the data plane according to the instructions from the intelligent remote control plane. However, this extraction of all intelligence from the FPGA comes at the cost of increased communication latency between the control plane and the data plane, resulting in a slower network reaction [18]. Therefore, some studies have proposed the concept of returning the control logic to the switch [18,19,20]. As heterogeneous architectures move toward tighter integration, FPGA manufacturers are beginning to integrate the CPU and FPGA resources to form a multiprocessor system on a chip (MPSoC) [21,22]. Therefore, the implementation of the control plane is no longer tied to “remote devices” but can also be implemented in the CPU of the MPSoC, thus reducing the latency of communication. The CPU and FPGA collaborate through this tightly coupled form and SDNs can be accelerated by a single chip.

In this paper, we propose an architecture with high performance, low communication latency, and flexibility for accelerating SDN based on the MPSoC platform. By taking advantage of the designed architecture, SDN can maintain its high speed, programmability, and flexibility. The main contributions of this paper are as follows:

- A CPU-FPGA cooperative high-performance architecture is proposed to accelerate SDNs, which can achieve a throughput of 100 Gbps. The control plane is implemented in the CPU, which is integrated with FPGA resources in the same MPSoC and the data plane is implemented in the FPGA logic. The communication between the two components is performed using Ethernet communication. To our knowledge, the architecture where the control plane and the data plane are integrated into the same MPSoC platform and communicate utilizing Ethernet is the first to be proposed.
- The architecture allows flexible and high-performance processing of packets to satisfy the needs of various network functions. The required network functions are easily deployed through the control plane.
- All the designed modules adopt the pipeline design and each module is connected through the AXI Stream interface. We also use a distributed RAM-based TCAM to implement a high-performance match table. The design runs at a clock frequency of 322 MHz and is capable of achieving 100 Gbps throughput performance.
- We implement the proposed flexible and high-performance architecture on an FPGA board. Use cases are also provided and evaluated to demonstrate the flexibility and high throughput.

The rest of the paper is organized as follows. The related work is discussed in Section 2. The designed architectures of the data plane and the control plane are detailed in Section 3 and Section 4, respectively. In Section 5, use cases are presented to illustrate the flexibility of the proposed architecture. The implementation and evaluation are shown in Section 6. Finally, the conclusion is given in Section 7.

## 2. Related Work

Some studies utilize FPGAs to design network switches to accelerate the data plane of SDN. Naous et al. [23] designed an Openflow switch for packet forwarding in the SDN data plane. Han et al. [24] also designed a switch based on FPGA. Although these designs accelerate the data plane, they all suffer from inflexibility. The tables in these designs are fixed and only a specific field, such as the destination IP address in the packet header, can be used as the matching field. Moreover, only a fixed set of actions can be performed on packets. If the network function changes, the whole design needs to be rewritten, which does not meet the flexibility requirements.

Many works utilize high-level language such as P4 to program the FPGA to accelerate networks. P4 is a protocol-independent high-level programming language [25]. The P4 compiler translates the P4 program to configure the target device. P4FPGA [26] is used to develop and evaluate data plane applications. It is a P4-to-FPGA compiler and runtime that first translates the P4 program into Bluespec [27] by the Bluespec compiler, and then the Bluespec code is compiled to Verilog. This means that this work depends on a commercial compiler. P4-NetFPGA [28] is based on the NetFPGA SUME board and Xilinx P4-SDNet compiler. The P4-SDNet compiler can convert a P4 program into an encrypted HDL module. By integrating this HDL module, users can customize the processing of network packets. Cao et al. [29] designed a framework for converting P4 programs into VHDL. In contrast to the methods mentioned above, instead of compiling P4 programs directly into HDL code, it provides a per-built template library and realizes the desired functions through these optimized VHDL templates. These designs using P4 to accelerate the network all have a disadvantage in that the generated HDL code still needs to be synthesized and implemented to generate the bitstream. The whole process may take several hours, which cannot meet the on-the-fly configuration requirements for the network.

Some studies aim to use SmartNICs to accelerate the processing of network packets as an alternative to traditional NICs. Corundum [30] is a high-performance, FPGA-based network interface card. It has a high-performance data path and a custom PCIe DMA engine that can support over 10,000 queues. However, it only implements simple NIC functions and does not offload other network functions. To accelerate network functions, Li et al. [31] proposed a reconfigurable network processing pipeline, which divides the processing of packets into several modules. They also proposed a module indexing mechanism that allows modules to be connected in any order, thus enabling programmability. However, when the design was evaluated using the test traffic generated by the IXIA tester, it only achieved an Ethernet rate of about 1 Gbps. FlexNIC [32] is a flexible network DMA interface that aims to reduce packet processing overheads. Users can install packet processing rules into the NIC. The NIC processes packets and then sends them to the host memory. They focus on the communication between the FPGA and the host, whereas our design implements the control plane on the on-chip CPU.

Table 1 compares our work with other hardware-based works in terms of the implemented device, flexibility, and throughput. FlexNIC [32] can be implemented with a NIC (i.e., ASIC) and can achieve high flexibility. However, the throughput of this design is relatively low. Hamadi et al. [33] accelerated an Open vSwitch (OVS) fast path based on a network processing unit (NPU), which achieved 20 Gbps, but its flexibility was poor due to the limitations of the NPU. FAS [34] accelerated SDN switches based on FPGA and achieved about 8 Gbps. Drawerpipe [31] is an FPGA-based reconfigurable packet processing pipeline that enables flexible packet processing. However, the throughput was low when tested with IXIA. As shown in Table 1, our work was able to achieve high performance and flexibility at the same time.

## 3. The Architecture of the Data Plane

In this section, we focus on the FPGA design that is used to accelerate the SDN data plane. First of all, a NIC shell for packet transmission and reception is implemented to enable the processing of network packets. Then, the pipeline architecture for processing network packets flexibly, which is placed in the user logic of the NIC shell, is presented.

### 3.1. The Architecture of the NIC Shell

**Figure 1:**
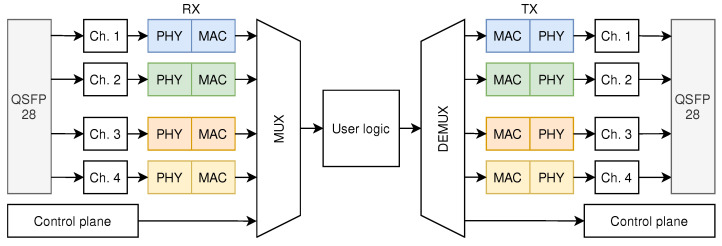
The architecture of the NIC shell.

The architecture of the NIC shell is shown in Figure 1. When packets are input from a QSFP28 port, they are first processed by the high-speed transceiver that performs clock recovery and serial-to-parallel conversion of the packets. Then, the packet frames are output in the form of an AXI4-Stream bus. Next, the packets from different channels and the control plane are fed to the MUX module, which combines the packets from different channels into a single data path. A user logic interface is implemented that allows the user to process the packets as desired. Packets are then input into the DEMUX module after being processed by the user logic. The DEMUX module is responsible for distributing packets to different channels or the control plane according to the control signal along with the packet. The control signal decides what action is to be performed on the packet. Actions include dropping the packet, outputting the packet to the destined channel, and outputting the packet to the control plane. The packets then enter the transmission path of the high-speed transceiver or the control plane.

### 3.2. The Architecture of the Pipeline

#### 3.2.1. Overview

**Figure 2:**
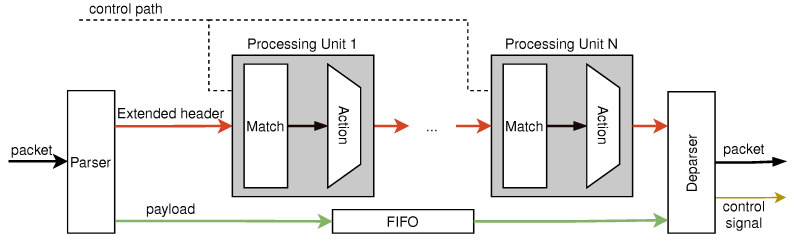
The reprogrammable match-action pipeline.

The implemented architecture is shown in Figure 2. This architecture is designed to be pipelined and is capable of performing a variety of network functions. The control plane configures the programmable pipeline through the control path. Packets first enter the parser module. The parser parses the packet header information, extracts the required header fields and metadata (such as the packet length, timestamp, etc.), and composes them into the extended header. In addition, the parser separates the packet header from the payload. The packet payload is cached by the FIFO, whereas the extended header enters the subsequent match-action pipeline for processing. The match-action pipeline consists of multiple programmable processing units. The programmable processing units are elaborately designed to achieve line-rate processing. When an extended header enters the processing unit, a field in the extended header is used as the matching field to search for the corresponding action that should be performed. If the extended header matches a rule, then the corresponding action will be performed. If the extended header does not match any rules, the extended header will be sent directly to the next processing unit. Then, the extended header is output to the deparser module after being processed by multiple processing units. The deparser combines the header and the payload to form a new packet and generates the control signal to determine whether the packet is forwarded, dropped, or uploaded to the control plane.

#### 3.2.2. The Parser and Deparser

The parser identifies the header type and extracts the required fields. A pipelined parser was designed in our architecture. The parsing process is divided into four stages. Each stage processes a layer of protocol and the relevant header fields are extracted to form the extended header. In the first stage, the Ethernet header is first parsed and the MAC addresses are extracted. The VLAN header is parsed in stage 2 if it exists and the next header type is identified. The IP header and the header of the layer 4 protocol are parsed in the next two stages, respectively. Finally, the extended header is output from the parser module.

The deparser module receives the extended header processed by the pipeline and the payload that is cached in the FIFO (PAY_FIFO). The deparser assembles the relevant fields of the extended header into a new packet header and then stores it in a FIFO (HED_FIFO). When the merge operation is performed, one header is retrieved from the HED_FIFO and one payload is retrieved from the PAY_FIFO and the two are combined into a new packet. In addition, the module generates the control signal of the packet based on the extended header such as outputting the packet to the Ethernet port, outputting the packet to the control plane, or dropping the packet.

#### 3.2.3. The Programmable Processing Unit

Each programmable processing unit is responsible for handling a part of the network function and is arranged as a pipeline to perform the entire network function. To realize the flexibility of network functions, it is necessary to achieve (1) The programmability of the matching field. A processing unit cannot only match one specific field but needs to be able to select one field of the extended header as the matching field according to the instruction of the control plane; (2) The programmability of the matching pattern. Different matching fields are matched in different ways. For example, we only care about the highest few bits of the IP address, whereas all the bits of the TCP port need to be matched. Three different matching patterns need to be implemented, that is, exact match (EM), mask match (MM), and longest prefix match (LPM); and (3) The programmability of the action. If each processing unit can only perform one kind of action, the network functions realized by the pipeline will be quite limited. The flexible processing of network packets can be achieved only by realizing the programmability of the matching fields, matching patterns, and actions in the processing unit.

**Figure 3:**
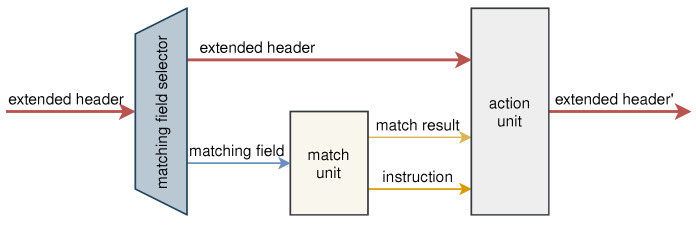
The architecture of the programmable processing unit.

As shown in Figure 3, the processing unit is composed of three parts, which are the matching field selector, match unit, and action unit. The control plane configures these three parts by sending instructions to make the processing unit perform the appropriate function. The matching field selector selects a certain field in the extended header for matching based on the instructions of the control plane, which enables the programmability of the matching field. The field is matched in the match unit, and the match result, together with the corresponding instruction, is output to the action unit. The action unit performs the corresponding action to process the extended header according to the instruction. After being processed, the extended header is output to the next processing unit.

**Figure 4:**
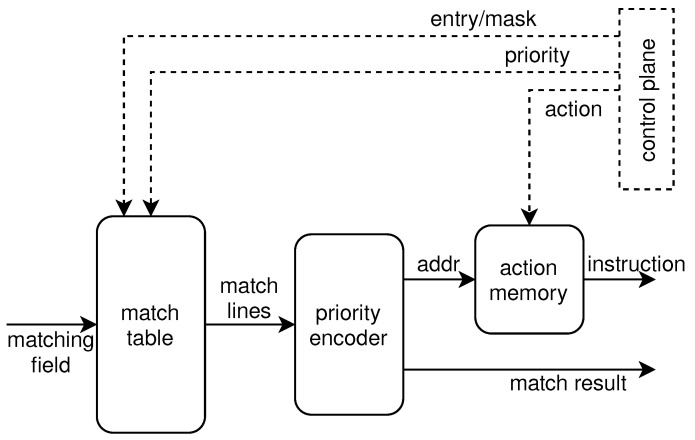
The design of the match unit.

The match unit is shown in Figure 4, including a match table, priority encoder, and an action memory. To achieve the programmability of the matching pattern, the match table is implemented by ternary content-addressable memory (TCAM). TCAM allows a matching state of don’t care, thus it can perform matching patterns such as EM, MM, and LPM. It also can perform one search operation per cycle, thus meeting the requirements of pipeline design to achieve high performance. Some designs use probabilistic data structures to implement match tables. Although this method guarantees that the error probability is under a certain threshold, it is not error-free. Incorrect lookup of table entries can lead to incorrect processing of packets, increasing network overhead. More importantly, the probabilistic data structure uses hash functions to represent a set of items and cannot support MM and LPM. The processing of the match unit is as follows. The matching field is first compared with the table entries stored in the match table. Each entry carries a priority and if multiple entries are hit, the priority encoder outputs the address of the hit entry with the highest priority. The address is then used to index the instructions stored in the action memory, which is implemented with the block RAM. The match result is also generated by the priority encoder; it indicates the matching field hits in at least one table entry. The instruction and the match result are then output to the action unit.

**Figure 5:**
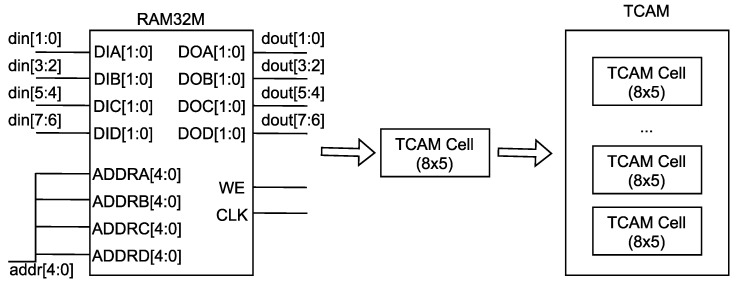
The design of the TCAM.

There is no dedicated TCAM resource in an FPGA. In order to achieve high-performance table lookup, we emulated the TCAM based on the distributed RAM. We used the RAM32M primitives of Xilinx devices to build the storage part. The RAM32M primitives were implemented using LUT resources. The structure of the RAM32M is shown in Figure 5. We connected four ADDR interfaces to configure the RAM32M as a RAM with a depth of 32 (2^5^) and a width of 8. The scheme to emulate the RAM-based TAM adopted the idea of transposition, i.e., the entry of TCAM is used as the address to index the RAM. Therefore, a RAM32M can emulate a TCAM cell that stores 8 entries with a width of 5. By combing multiple TCAM cells, we obtained the TCAM of the desired width and depth. The RAM32M can be accessed in every cycle, so the implemented TCAM was able to perform the match operation in every cycle, thus enabling high-performance table lookup.

**Figure 6:**
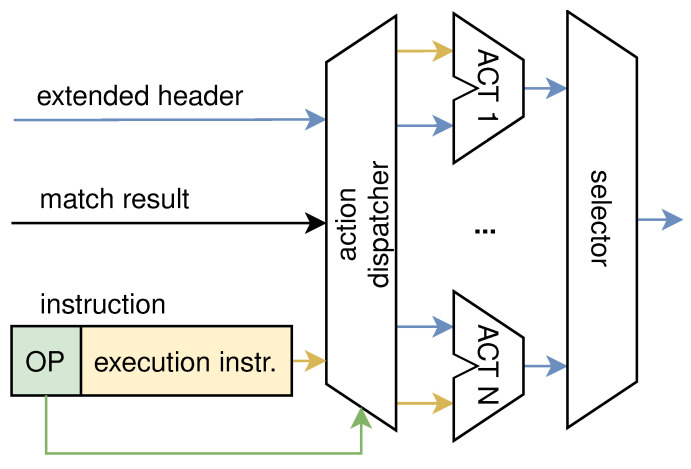
The design of the action unit.

The action unit is shown in Figure 6. In traditional architectures, the pipeline architecture can only perform a fixed series of actions that serve a specific network function. For example, modifying the MAC address is performed in the first stage and deleting the VLAN header is performed in the second stage. In each stage, only the fixed action is performed. The function and sequence of these actions in the architecture are fixed and cannot be changed. Therefore, these architectures are not flexible. The action dispatcher was designed to enable the programmability of the action; therefore, each processing unit was no longer limited to one particular type of action. The processing of the action unit is as follows. The instruction output from the match unit consists of two parts: the option field and the execution instruction. According to the option field, the action dispatcher distributes the extended header to different execution units, each of them executing one type of action. The execution unit processes the extended header according to the execution instruction, including modifying header fields, inserting header fields, modifying packet metadata, etc. For example, if a VLAN ID is intended to be modified, the execution unit will retrieve the VLAN ID from the execution instruction and insert it into the extended header. After being processed by the action, the extended header is output through the selector.

### 3.3. The Challenge of Achieving 100 Gbps Performance

Implementing the data plane in software is the most popular scheme at present. However, it is difficult for these software solutions to achieve high throughput. First, the performance of table lookup is poor. In a 100 Gbps network, the packet rate for transmitting a small 64-byte packet is 148.8 Mpps. Even with the most efficient exact match, it is difficult to achieve a lookup rate of 100 M lookups per second using a CPU. Second, the required actions need to be performed on packets in the data plane. These actions involve intensive memory read and write operations. This further reduces the throughput and increases the processing latency.

To address the low performance of the software method, we made some optimizations in the proposed hardware architecture. First, there was no dedicated TCAM resource in the FPGA so we implemented a high-performance TCAM to implement the three matching patterns. We emulated a TCAM based on distributed RAM, which can perform a search operation in every cycle. The clock frequency used in our design was 322 MHz so a search operation can be performed 322 M times per second, which is greater than the search performance required for a 100 Gbps network. Second, we optimized each action. The execution time of each action was one clock cycle so the processing delay was minimized. Third, the architecture employed a pipeline design that is capable of processing packets every clock cycle. The clock frequency in the design was 322 MHz, and the width of the bus was 512 bits. For 64B small packets, the packet rate was 148.8 Mpps. If a 512-bit bus is used, to achieve a throughput of 100 Gbps, the minimum clock frequency to be achieved is 148.8 MHz. Therefore, the design can meet the requirements of 100 Gbps throughput.

## 4. The Architecture of the Control Plane

The separation of the control plane from the data plane is one of the characteristics of SDN. However, the communication between the local data plane and the remote control plane introduces a certain latency, which causes the network to be slower in response. This situation is aggravated when the control plane network experiences congestion. Implementing the control plane on the on-chip CPU allows the on-chip CPU to cooperate with the FPGA logic, thus saving the overhead of the communication between the control plane and the data plane.

### 4.1. The Architecture Overview

**Figure 7:**
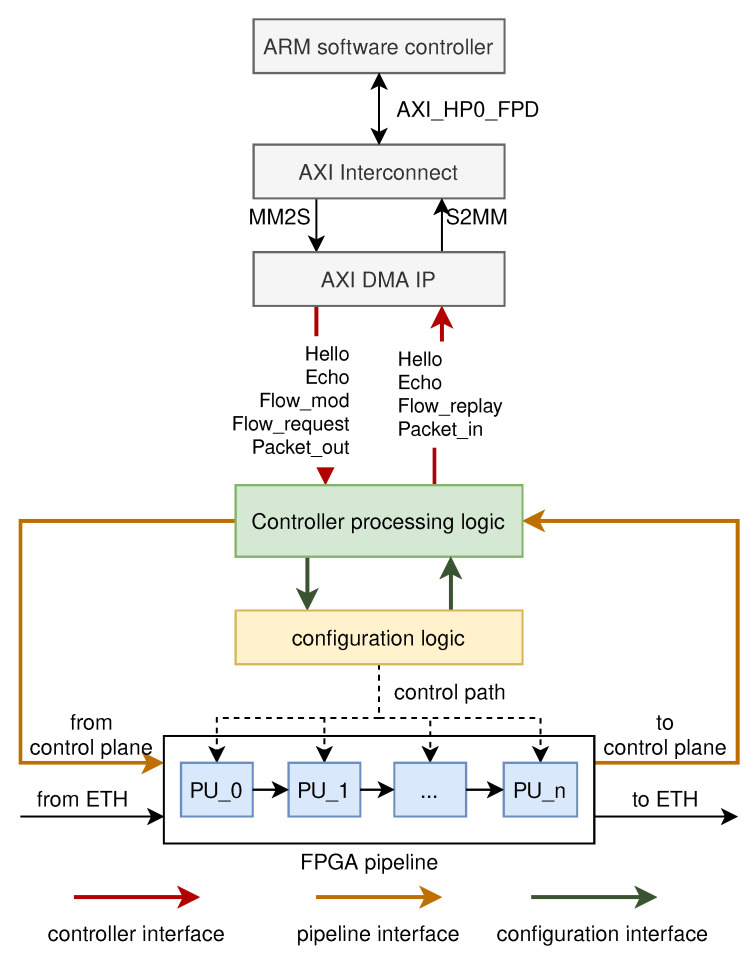
The communication between the control plane and the data plane.

The control plane is located in the on-chip CPU, and the data plane is located in the FPGA logic. To realize the cooperation between the control plane and the data plane, it is necessary to realize efficient communication between the on-chip CPU and the FPGA logic. In our implementation, the communication between the two components is performed by means of Ethernet communication. Figure 7 shows the overview of the communication between the control plane and the data plane. The communication between the control plane and the data plane is performed through the message packet. The message packet is a UDP packet that is transmitted at high speeds through the AXI_HP0_FPD interface of the ARM software controller. The interface is connected via the AXI Interconnect IP to the stream-to-memory-mapped (S2MM) interface and the memory-mapped-to-stream (MM2S) interface of the AXI DMA IP. Finally, the message packet transferred through the control plane and the data plane is output as an AXI4-Stream bus to the controller processing logic.

Table 2 shows part of a message packet. The types of message packets are the handshake message packet, the control message packet, and the data message packet. The controller processing logic is responsible for processing these message packets, as shown in Figure 7. The handshake messages are used to establish and maintain communication between the control plane and the data plane. When the controller processing logic receives a handshake message packet, it replies with a handshake message packet through the controller interface to establish and maintain communication between the data plane and the control plane. The control message packet is used to configure the processing units in the pipeline to realize the flexibility of the network functions, for example, configuring the type of matching field; adding, deleting, and modifying table entries in the match table; setting the actions of each processing unit, etc. The control message packet is processed as follows. The controller processing logic parses the packet to obtain the instructions. The instruction contains a sequence number for describing the processing unit to be configured and specific configuration instructions (such as configuring the matching field, actions, etc.). The configuration interface distributes the configuration instructions to the corresponding processing unit according to the sequence number. The data message packet is used for the packet transmission between the control plane and the data plane. For packets sent from the data plane to the ARM software controller, the controller processing logic receives these packets from the pipeline interface and sends them to the ARM software controller through the controller interface after processing. For packets sent from the ARM software controller to the data plane, the process is reversed.

### 4.2. The Controller Processing Logic

The controller processing logic is the bridge for communication between the control plane and the data plane. It is responsible for parsing, encapsulating, and delivering the message packets between the control plane and the data plane. It also maintains the communication connection between the control plane and the data plane.

As shown in Figure 7, the controller processing logic mainly has three groups of interfaces: the controller interface, the configuration interface, and the pipeline interface. The controller interface is used for the transmission of message packets between the ARM software controller and the data plane. The configuration interface is responsible for sending instructions parsed from the control message packet to the configuration logic. The pipeline interface connects the pipeline in the data plane and the controller processing logic. When packets need to be transmitted between the control plane and the data plane (such as Packet_in and Packet_out), the packets are transmitted through the pipeline interface.

In the controller processing logic, two cores handle message packets from the controller interface and packets from the pipeline interface, respectively. For packets from the controller interface, the filtering module first filters them based on the IP address and port, and only receives message packets; other packets or wrong message packets are directly discarded. The core1 module processes the message packets. For message packets to configure the data plane, core1 parses the packets and sends instructions to the configuration interface. For message packets that need to reply (such as establishing a connection, replaying a Flow_reply message to a Flow_request message), core1 generates the content of the message to be replied to and sends it to the assembler module for assembly. The assembled message is encapsulated into an Ethernet packet in the eth_ip_udp module and cached in the FIFO. Finally, the message packet is sent to the ARM software controller through the controller interface. For data message packets from the control plane (such as Packet_out), the data message packets are decapsulated and directly sent out through the pipeline interface.

When the data plane receives a packet that it does not know what to do with, the packet should be uploaded to the control plane. The data plane first sends the data packet through the pipeline interface to the controller processing logic. For controller processing logic, packets received from the pipeline interface need to be uploaded to the control plane and core2 is responsible for processing these packets. First, these packets are stored in the FIFO for buffering; core2 reads them and generates the message header. The assembler assembles the message. The eth_ip_udp encapsulates the messages into Ethernet packets. Then, the packets are stored in FIFO and are sent to the controller interface.

## 5. Case Study

To illustrate the flexibility of the designed architecture, we implemented some applications. These network functions can be configured quickly and efficiently through the control plane, allowing researchers to focus on the core functional logic and achieve rapid development.

**Figure 8:**
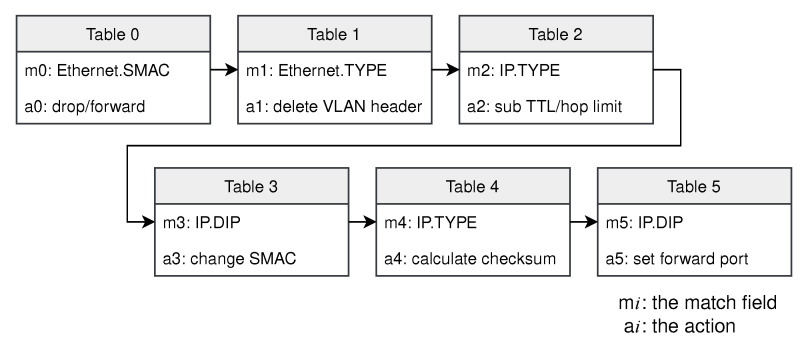
The match-action tables of the L2/L3 switch.

L2/L3 Switch. Switches with legacy architectures control the forwarding behavior using algorithms built into the hardware and it is therefore difficult to accommodate changes in the network. We offload the function of the switch to the proposed architecture. The parser mainly extracts the following fields of the packets: Ethernet type, source and destination MAC addresses, and source and destination IP addresses. The match-action tables implemented in the pipeline are shown in Figure 8. First, the source MAC address is examined. Packets with a multicast source MAC address are not valid and should be dropped. Then, it determines whether the packet has a VLAN header based on the Ethernet type. If so, the VLAN header is removed. Then, the pipeline recalculates the TTL for IPv4 packets or the hop limit for IPv6 packets. If the TTL or the hop limit is 0, the packet will be dropped. Next, the source MAC address is changed and the checksum is recalculated. Finally, the packet is forwarded from the corresponding port according to the IP address.

Packet Filtering. Packet filtering discards specific flows according to pre-defined filtering rules, thereby enabling access control of network packets. The packet filtering is offloaded to the proposed architecture. When a packet enters the filter, the filter first classifies the packet, usually based on the 5-tuple classification. Then, the filter compares the 5-tuple classification with the pre-defined rules to decide whether the packet should be passed or dropped. If the packet has the desired IP and port, the packet will be passed. The control plane sends the required filtering rules to the data plane and the corresponding action (forward, upload, or drop) can be set for each rule to realize packet filtering.

Applications in real networks. Our institute is working on a new information-centric networking (ICN) architecture. In the original network architecture, packet forwarding was handled by servers, which limited the overall performance of the network. The performance was about 20 Gbps. To improve the overall performance of the network, our design was applied to the new network architecture to accelerate packet forwarding. A significant performance improvement was achieved over the original architecture.

## 6. Implementation and Evaluation

The designed architecture was implemented in an FPGA board with a Xilinx XCZU19EG-2FFVC1760E. We used this FPGA because it is suitable for high-performance network packet processing and has sufficient resources to support our proposed 100 Gbps high-performance architecture. This FPGA integrated a 64-bit quad-core Arm Cortex-A53 and dual-core Arm Cortex-R5F with rich features to satisfy the requirements of the control plane. A QSFP28 Ethernet transceiver composed of four independent 25 Gbps transmit and receive channels was used to provide the 100 Gbps Ethernet link. The design ran at a clock frequency of 322 MHz with a 512-bit AXI4-Stream bus; hence, the total bandwidth can satisfy the performance requirement of 100 GbE. The architecture was implemented by carefully designed Verilog code to ensure high performance.

The test environment is shown below. A Dell R730 commodity server was used for comparative testing. The server was equipped with two Intel Xeon E5-2609 v3 1.9 GHz CPUs, both with 6 cores, and 32 GB DDR4 memory. An Intel E810-C 100G NIC was installed in the PCI Express 3.0 × 16 slot and was configured in 4 × 25 G mode. The server ran on CentOS 7.9 with kernel 3.10.0. To test the designed architecture, the IXIA tester connected to the NIC or FPGA via a fiber optic cable and served as a packet generator.

The remainder of this section is as follows. In Section 6.1, we first evaluate the impact of multiple pipeline stages on the performance of the designed architecture, including throughput and latency. In Section 6.2 and Section 6.3, we evaluate the performance of the two network applications introduced in Section 5 to illustrate that the proposed architecture achieves flexibility and high-performance requirements. We evaluate the communication latency between the control plane and the data plane in Section 6.4. The resource usage of the designed pipeline architecture is evaluated in Section 6.5.

### 6.1. Evaluation of Multiple Pipeline Stages

In this part, to illustrate the impact of the number of pipeline stages on performance, a software application based on the DPDK was built for comparison. In this application, one pipeline was first built and processed with one CPU core to illustrate the worst-case performance variation. The pipeline had multiple stages and each stage only performed the longest prefix match on the destination IP address. The matched packets were sent to the next pipeline stage and forwarded to the corresponding port based on the destination IP address at the last stage. The FPGA pipeline was also configured to perform the same function. The IXIA tester generated test traffic with a packet length of 64B to 1024B. The test traffic was composed of multiple flows. After being processed by the FPGA or CPU, the test traffic was forwarded back to IXIA.

**Figure 9:**
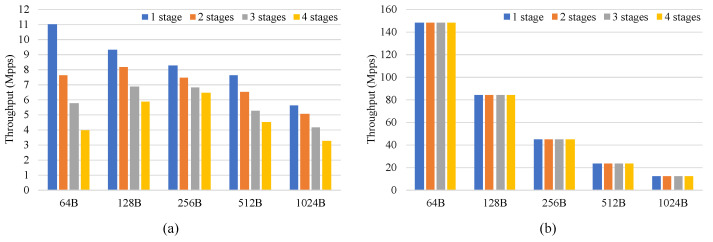
The throughput of the (**a**) CPU and (**b**) FPGA.

Figure 9 shows the throughput of the CPU and FPGA. When the number of pipeline stages was constant, the FPGA processed packets at line rate for different packets, whereas the performance of the CPU varied with different packet lengths. The throughput that the CPU achieved was about 5.63 Mpps (47 Gbps) when processing 1024B packets, and the FPGA achieved higher throughput than the CPU for different packet lengths. This shows that the design can achieve high performance for different packet lengths. When the packet length was constant, the FPGA maintained a constant throughput for different pipeline stages and processed packets at line rate. However, as the number of pipeline stages increased, the performance of the CPU showed a degradation. This decreasing trend held for pipelines larger than four stages. This indicates that the designed architecture can maintain high throughput for a different number of pipeline stages.

**Figure 10:**
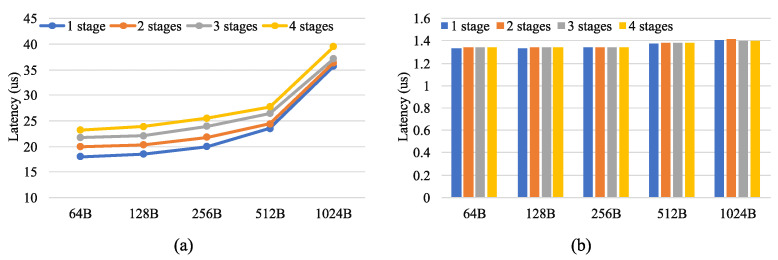
The latency of the (**a**) CPU and (**b**) FPGA.

Figure 10 compares the latency of the CPU and FPGA. Figure 10a shows the latency of the CPU and Figure 10b shows the latency of the FPGA. The latency of the CPU increased with the number of pipeline stages and packet length, whereas the latency of the FPGA was relatively constant. In addition, the latency of the FPGA was about 1.3us, which was significantly lower than that of the CPU for the different packet lengths and the number of pipeline stages.

**Figure 11:**
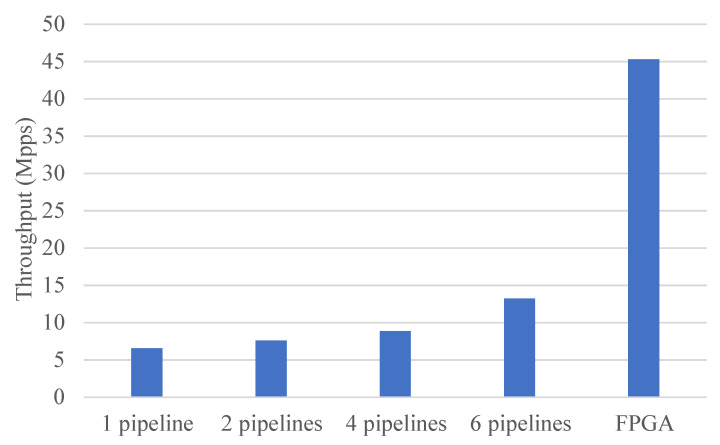
The throughput comparison of the CPU and FPGA.

Next, by evaluating the impact of multiple CPU cores on performance, it was shown that using the FPGA can effectively save CPU resources. Different numbers of pipelines were built, each pipeline contained four stages, and different pipelines ran on different CPU cores. For optimal performance, the used CPUs and NIC were on the same NUMA node. Figure 11 shows the throughput comparison of the CPU and FPGA when IXIA sent test traffic with a length of 256B. It shows that as the number of pipelines increased, the throughput of the CPU also improved. When the number of pipelines was six (i.e., when six CPU cores were used), the maximum performance that the CPU achieved was about 13.15 Mpps (29.04 Gpbs). The FPGA processed the packets at line rate and its throughput was significantly higher than the CPU’s throughput. Because the poll-mode driver (PMD) was used to handle the packet I/O of the DPDK interfaces, 50% of the total 12 CPU cores were fully occupied the entire time. By accelerating the packet processing with the designed architecture, performance was significantly improved and a lot of CPU resources were saved.

### 6.2. Evaluation of L2/L3 Switch

The L2/L3 switch described in Section 5 was evaluated. To illustrate the performance gain achieved by using the designed architecture to accelerate packet forwarding, OVS with DPDK was used as a software implementation for comparison. In this evaluation, the Open vSwitch 2.13.6 and DPDK 19.11.6 were used. The parameters were carefully tuned for optimal performance including core affinity, huge pages, poll-mode driver threads, etc. The IXIA tester generated packets with different lengths, which were processed by the OVS or FPGA and forwarded back to the IXIA tester.

**Figure 12:**
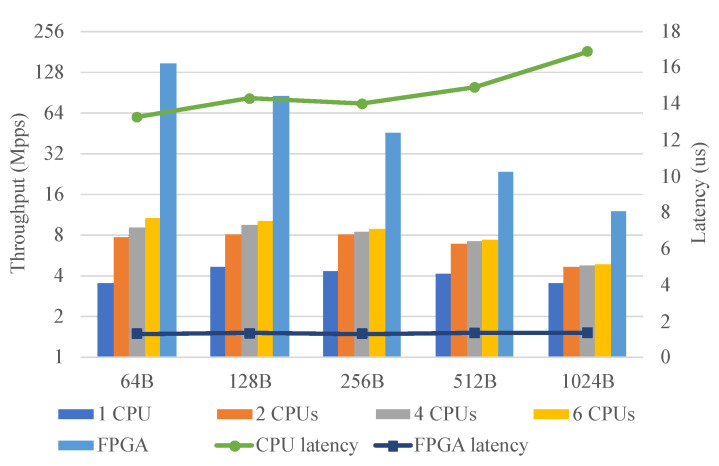
L2/L3 switch. The throughput and latency of the CPU and FPGA. Bars show the throughput, lines show the latency.

Figure 12 shows the throughput and latency of the CPU (OVS) and FPGA. The bars show the throughput of the CPU and FPGA and the lines show the latency of the CPU and FPGA. As the number of CPU cores used increased, the throughput of the CPU also increased. The performance of the CPU reached the maximum when six CPU cores were fully occupied. In contrast, the throughput of the FPGA reached line rate for any packet length. When processing 64B packets, the performance of the FPGA was approximately 13–40 times that of the CPU. In addition, the latency of the FPGA was much lower than that of the CPU at any packet length and was reduced by about 90%.

### 6.3. Evaluation of Packet Filtering

**Figure 13:**
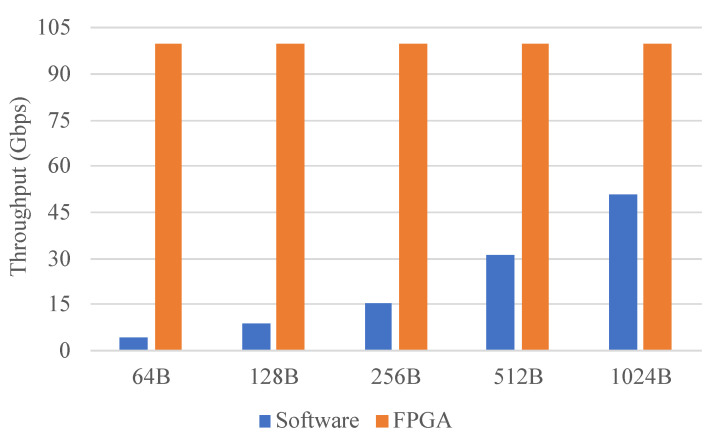
Packet filtering. The throughput of the software and the FPGA.

The performance of the packet filtering described in Section 5 was evaluated. As a comparison, we also implemented software packet filtering using DPDK. IXIA was used to generate test traffic of various lengths. To evaluate the maximum performance that the packet filtering can achieve, all packets sent by IXIA will hit the rules and be forwarded back. Figure 13 shows the throughput of the software method and the FPGA. The FPGA achieved line rate for any packet length but the throughput of the software method was related to the packet length and far from line rate. This shows that the packet filtering implemented by the designed architecture can meet the high-performance requirement.

### 6.4. Communication Latency

**Figure 14:**
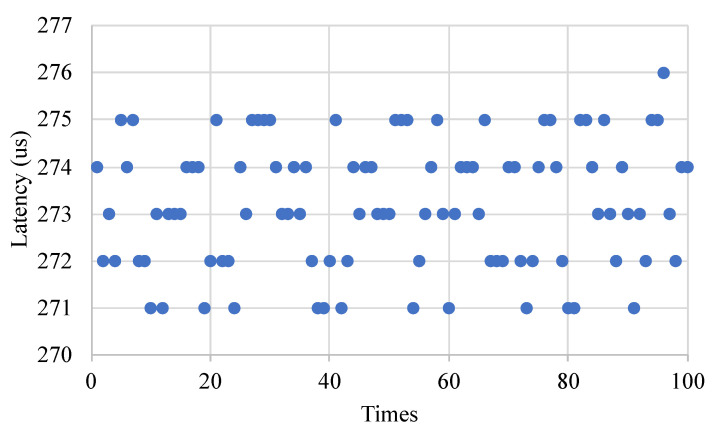
The round-trip communication latency between the control plane and the data plane.

The round-trip communication latency between the control plane and the data plane was evaluated. To measure the communication latency between the control plane and the data plane, we sent a Flow_request message packet from the control plane to the data plane. The data plane returned the Flow_reply message packets to the control plane after receiving the Flow_request message packets. The round-trip latency was obtained by calculating the timestamp of the corresponding message packets. Figure 14 shows the round-trip communication latency between the control plane and the data plane. We measured 100 sets of data and the communication delay was about 273 μs.

### 6.5. Resource Utilization

Resource utilization is related to the number of stages in the pipeline and the size of the matching table in each stage. Fewer table entries with fewer widths will consume fewer resources. In this evaluation, we used match tables with a width of 128 bits, action memories with a width of 160 bits, and a total number of table entries of 2048. Table 3 shows the resource utilization, which mainly included lookup tables (LUTs), flip-flops (FFs), and memory resources. The block RAMs (BRAMs) were mainly used by action memories and FIFOs. The LUT resource consumption was high because there was no dedicated TCAM resource in the FPGA so the TCAM was implemented based on distributed RAM. In addition, many solutions use block RAM to implement TCAM [35,36] so the resource usage of LUT and block RAM can be balanced by combining the two solutions.

Table 3 also compares our work with other related works. FAS [34] implemented an SDN switch based on an FPGA. DrawerPipe [31] designed a reconfigurable network processing pipeline. Blueswitch [24] implemented the related functions of data plane forwarding. Compared with the other works, our work used more LUT resources because the TCAM in our work was based on distributed RAM. As shown in Table 3, the power consumption of our work was about 53.31 W, which was estimated by Vivado after implementation. The power consumption included the FPGA logic and the on-chip CPU. The share of static power consumption was 13% and the share of dynamic power consumption was 87%. The throughput of our work was able to achieve 100 Gbps, which is higher than the performance of other works.

## 7. Conclusions

In this paper, we propose a high-performance and flexible architecture for accelerating SDN on an MPSoC platform that provides sufficient flexibility to support various network functions while achieving high performance at 100 Gbps. The design is capable of achieving better performance than CPUs and is more flexible than ASICs. The control plane is implemented in the on-chip CPU and the data plane is implemented in the FPGA logic resources, providing the flexibility to accelerate packet processing. The required network functions are easily deployed through the control plane. By taking advantage of the flexibility and high performance of the designed architecture to accelerate SDN networks, a significant amount of valuable CPU resources can be saved. The results of the measurements show that the performance of the designed architecture is significantly improved compared to the performance of the CPU, and when the designed architecture performs different network functions, it is still able to maintain high performance, which can meet the requirements of future high-speed networks.

## Figures and Tables

**Table 1:** Comparison with other related works.

Work	Device	Flexibility	Throughput
FlexNIC [32]	ASIC	High	20 Gbps
Hamadi et al. [33]	NPU	Low	20 Gbps
FAS [34]	FPGA	High	8 Gbps
Drawerpipe [31]	FPGA	High	1 Gbps
Our work	FPGA	High	100 Gbps

**Table 2:** Part of a message packet.

Category	Type	Direction	Description
Handshake message	Hello	CPU to FPGA; FPGA to CPU	Establishes the connection between the control plane and the data plane.
	Echo	CPU to FPGA; FPGA to CPU	The heartbeat signal that is used to maintain the connection.
Control message	Flow_mod	CPU to FPGA	Used to configure programming processing units.
	Flow_request	CPU to FPGA	Used to obtain the configuration information of programmable processing units.
	Flow_reply	FPGA to CPU	Used to send the configuration information of the corresponding programmable processing unit to the control plane.
Data message	Packet_in	FPGA to CPU	Used to send a packet from the data plane to the control plane.
	Packet_out	CPU to FPGA	Used to send a packet from the control plane to the data plane.

**Table 3:** The resource utilization.

Work	Device	LUT/ALM	FF/Register	BRAM/URAM	Throughput	Power
DrawerPipe [31]	Xilinx Zynq-7000 SoC	6.55%	6.55%	35.74%	1 Gbps	-
FAS [34]	Altera EP4SGX180	21%	11%	11%	8 Gbps	-
Blueswitch [24]	Xilinx Virtex-5 TX240T	11%	28%	-	10 Gbps	-
Our Work	Xilinx XCZU19EG	41%	10.43%	35.72%	100 Gbps	53.31 W

## Data Availability

All the necessary data are included in the article.
